# Burnout, Mental Health, and Workplace Characteristics: Contributors and Protective Factors Associated With Suicidal Ideation in High‐Risk Nurses

**DOI:** 10.1111/wvn.70042

**Published:** 2025-06-05

**Authors:** Bernadette Mazurek Melnyk, Judy E. Davidson, Sharon Tucker, Alai Tan, Andreanna Pavan Hsieh, Andrea Cooper, Cora Mayfield, Jacqueline Hoying

**Affiliations:** ^1^ COPE2Thrive LLC Powell Ohio USA; ^2^ EBP Solutions LLC Powell Ohio USA; ^3^ The Ohio State University College of Nursing Columbus Ohio USA; ^4^ University of California San Diego Health San Diego California USA; ^5^ University of Central Florida Orlando Florida USA

**Keywords:** burnout, job satisfaction, mattering, nurses, suicide, workplace

## Abstract

**Background:**

A call for action has been issued nationwide to prevent suicide among nurses. An increased understanding of contributing and protective factors associated with suicidal ideation in nurses is needed to implement preventive measures. Factors needing exploration include nurses' burnout, mental well‐being, physical health, and workplace characteristics.

**Aims:**

This study aimed to determine factors associated with suicidal ideation in 501 moderate‐to‐high‐risk nurses, including their mental health, level of burnout, health‐related personal beliefs, healthy lifestyle behaviors, and workplace characteristics.

**Methods:**

A descriptive, cross‐sectional correlational study was conducted on baseline survey data that was completed before the nurses were randomized to one of two interventions as part of their participation in a randomized controlled trial investigating the efficacy of a combined mental health screening program and cognitive‐behavioral skills building intervention versus a screening program alone. Nurses were recruited from across the United States via email. Only nurses identified with moderate‐to‐high‐risk adverse mental health outcomes, including suicidal ideation, were included. The survey used valid and reliable measures to assess burnout, anxiety, depression, suicidal ideation, post‐traumatic stress, healthy lifestyle behaviors, health‐related personal beliefs, resilience, job satisfaction, self‐perceived mattering to the workplace, and intent to leave. Bivariate tests were performed.

**Results:**

Burnout, anxiety, depression, and post‐traumatic stress were individually correlated with increased odds of suicidal ideation, as were nurses working 12‐h shifts and those who reported an intent to leave their jobs. Protective factors against suicidal ideation included resilience, positive health‐related personal beliefs, healthy lifestyle behaviors, job satisfaction, and workplace mattering.

**Linking Action to Evidence:**

There is an urgent need for policies and implementation of evidence‐based interventions to address mental health issues in nurses to ultimately prevent suicide. Burnout should be considered as a possible precursor to serious adverse mental health problems and not just an operational retention issue. Leaders need to invest in resources to enhance nurses' mental health, fix system problems that are at the root cause of burnout, routinely recognize employees for their excellent work, and communicate that they matter. Leaders should listen carefully to their nurses, prioritize their ideas for impactful change, and appreciate those who contribute to improving culture and caring practices.

## 1 Background

Suicide is a leading cause of death in the United States (US), with one person dying by suicide every 11 min (Centers for Disease Control and Prevention [CDC] 2025). Contributing factors to suicide at the individual level include mental health conditions (e.g., depression, anxiety, post‐traumatic stress), previous suicide attempts, job or financial issues, and substance use (CDC 2024). Some studies suggest that burnout may also play a role in suicidal ideation (Kelsey et al. 2021; Oh et al. 2023; Zisook et al. 2022). Understanding suicidal ideation in the context of burnout is complex as depression often coexists (Menon et al. 2020). Due to increased rates of burnout and mental health conditions in the healthcare workforce, there is a growing concern over suicide and suicidal ideation among this occupational population, especially in nurses (Kelsey et al. 2021; Li et al. 2024).

A national retrospective cohort study using 2005–2016 data from the National Violent Death Reporting System of nurse suicide in the United States established that nurses were at greater risk of suicide than the general population (Davidson et al. 2020). An update of 2017–2021 data found that female nurses were at higher risk of suicide than other females, whereas male nurses had comparable rates to male non‐nurses (Davidson et al. 2024). Suicide is a catastrophic event, and over 70% of individuals who die by suicide do not receive needed support services prior to their death (Ku et al. 2021). Roughly 1 in 18 nurses report having suicidal ideation in the past year, which is higher than non‐nurses, and these impacted nurses are less likely to seek help (Kelsey et al. 2021). To prevent suicide in nurses, risk factors must be understood and recognized.

Depression is a widely recognized risk for suicidal thoughts and behaviors, with burnout playing a contributing role (Kelsey et al. 2021; Oh et al. 2023; Zisook et al. 2022). In a cross‐sectional study with 771 critical care nurses, 39.5% had depressive symptoms (Melnyk et al. 2021). Another cross‐sectional study with 125 nurse managers found smaller rates of depression, 15% (Melnyk, Chenot, et al. 2024), suggesting that the prevalence may vary depending on the nurse role.

Burnout continues to be a dominating concern in the nursing profession, with a professional white paper indicating that 65% of nurses report burnout (*Beyond the Bedside* 2025). Top stressors that contribute to nurses' poor mental health include short staffing, insufficient pay and benefits, lack of leadership support, high‐pressure environments, verbal or physical abuse from patients, numerous bureaucratic tasks that take time away from caring for patients, and a feeling that they do not matter (*Beyond the Bedside* 2025; Melnyk, Strait, et al. 2023; Melnyk, Chenot, et al. 2024). Other modifiable risk factors for nurses include shift work and healthy lifestyle behaviors (Melnyk et al. 2021; Yang et al. 2024). Prior studies have indicated that workplace well‐being support for nurses, including perceptions of mattering to the organization and having a supportive organizational wellness culture, result in less positive screenings for depression, anxiety, stress, and burnout (Melnyk et al. 2022; Melnyk, Hsieh, et al. 2023; Melnyk, Strait, et al. 2023; Melnyk, Chenot, et al. 2024). The work environment appears to play an important role in nurses' mental health and highlights an area for improvement in terms of suicide prevention.

### 1.1 Aims

Currently, there is a paucity of research specifically investigating contributors and protective factors associated with suicidal ideation in nurses identified with moderate‐to‐high‐risk adverse mental health outcomes, including burnout, anxiety, depression, post‐traumatic stress, resilience, health‐related personal beliefs, healthy lifestyle behaviors, and workplace characteristics such as shift length, job satisfaction, self‐perceived mattering to workplace, and intent to leave.

Therefore, this study aimed to determine which of these factors are associated with suicidal ideation and which are protective in nurses with moderate‐to‐high‐risk adverse mental health outcomes.

## 2 Methods

### 2.1 Sample

Five hundred and one nurses were enrolled from a national sample via a recruitment email sent by Healthy Nurse Healthy Nation, their employing health system, or professional nursing organization. Random and purposive sampling was used to assure distribution nationally.

### 2.2 Study Design

This study used a descriptive, correlational cross‐sectional design. Data were obtained from a baseline survey completed before the nurses were randomized to one of two interventions as part of their participation in a randomized controlled trial investigating the efficacy of a combined mental health screening program and cognitive‐behavioral skills building intervention versus a screening program alone. Specifics about the RCT's methods and instruments are previously published (Melnyk, Davidson, et al. 2024). Survey data were collected via REDCap (i.e., Research Electronic Data Capture), a secure, web‐based software platform designed to support data capture (REDCap, n.d.).

The study was approved by the first author's Institutional Review Board and registered at the US National Institutes of Health #NCT05582343. After providing consent, participants were screened using a modified version of the Interactive Screening Program (i.e., mISP) developed by the American Foundation for Suicide Prevention (AFSP, n.d.; Melnyk, Davidson, et al. 2024). Those who screened at moderate‐to‐high risk for adverse mental health outcomes, including suicidal ideation, were then provided a link to complete a survey that included demographic questions, valid and reliable mental health measures (suicidal ideation, burnout, anxiety, depression, post‐traumatic stress, resilience), healthy lifestyle beliefs, healthy lifestyle behaviors, and questions concerning workplace characteristics (shift length, job satisfaction, perceptions of mattering to the workplace, intent to leave their job in next year).

### 2.3 Measures

Details of the valid and reliable instruments used to measure burnout, anxiety, depression, post‐traumatic stress, job satisfaction, healthy lifestyle behaviors, and healthy lifestyle beliefs have been described in detail elsewhere (Melnyk, Davidson, et al. 2024). Table 1 lists the specific measures and their corresponding reliability, including details for resilience and mattering. Item‐9 on the Patient Health Questionnaire‐9 (PHQ‐9) “Thoughts that you would be better off dead or of hurting yourself in some way?” was used as the anchor for all analyses regarding suicidal ideation (Zhang et al. 2025). The Patient Health Questionnaire‐8 (PHQ‐8) was used as a measure of depression without suicidal ideation (Kroenke et al. 2009). The PHQ‐8 excludes item 9 from the PHQ‐9.

**TABLE 1:** Study outcome variables and their corresponding measures.

Outcome	Measure	Cronbach's alpha	Reference
Demographics	Age, gender, race, ethnicity, marital status, shift length	Not applicable	Not applicable
Burnout	Single‐item burnout question Score range = 1 (no symptoms of burnout) to 5 (completely burned out)	Not applicable AUC = 0.93	Dolan, E. D., Mohr, D., Lempa, M., Joos, S., Fihn, S. D., Nelson, K. M., & Helfrich, C. D. (2015). Using a single item to measure burnout in primary care staff: A psychometric evaluation. *Journal of General Internal Medicine*, *30*(5), 582–587. https://doi.org/10.1007/s11606‐014‐3112‐6
Suicidal ideation	Item 9, PHQ‐9 Score range = 0 (not at all) to 3 (most or all the time)	Not applicable	Zhang, S., Zisook, S., Davidson, J., Shapiro, D., & Doran, N. (2025). Suicidal thoughts and behaviors among health care trainees, staff and faculty at an academic medical center. *Journal of Clinical Medicine*, *14*(2), 574. https://doi.org/10.3390/jcm14020574
Depression without suicidality	PHQ‐8 Score range = 0–24 ≥ 5 = mild to severe depression	0.89	Kroenke, K., Strine, T. W., Spitzer, R. L., Williams, J. B., Berry, J. T., & Mokdad, A. H. (2009). The PHQ‐8 as a measure of current depression in the general population. *Journal of Affective Disorders*, *114*(1–3), 163–173. https://doi.org/10.1016/j.jad.2008.06.026 Shin, C., Lee, S. H., Han, K. M., Yoon, H. K., & Han, C. (2019). Comparison of the usefulness of the PHQ‐8 and PHQ‐9 for screening for major depressive disorder: Analysis of psychiatric outpatient data. *Psychiatry Investigation*, *16*(4), 300–305. https://doi.org/10.30773/pi.2019.02.01
Anxiety	GAD‐7 Score range = 0–21 ≥ 5 = mild to severe anxiety	Above 0.85	Löwe, B., Decker, O., Müller, S., Brähler, E., Schellberg, D., Herzog, W., & Herzberg, P. Y. (2008). Validation and standardization of the Generalized Anxiety Disorder Screener (GAD‐7) in the general population. *Medical Care*, *46*(3), 266–274. https://doi.org/10.1097/MLR.0b013e318160d093
Post‐traumatic stress disorder	PC‐PTSD‐5 Score range = 0–5 ≥ 3 = probable PTSD	Not applicable AUC + = 0.941	Prins, A., Bovin, M. J., Smolenski, D. J., Marx, B. P., Kimerling, R., Jenkins‐Guarnieri, M. A., Kaloupek, D. G., Schnurr, P. P., Kaiser, A. P., Leyva, Y. E., & Tiet, Q. Q. (2016). The Primary Care PTSD Screen for DSM‐5 (PC‐PTSD‐5): Development and evaluation within a veteran primary care sample. *Journal of General Internal Medicine*, *31*(10), 1206–1211. https://doi.org/10.1007/s11606‐016‐3703‐5
Job Satisfaction Scale	JSS Score range = 7–35 Higher scores indicate higher satisfaction	0.80 and above	Price, J. L., & Mueller, C. W. (1981). Professional turnover: The case of nurses. *Health System Management*, 15, 1–160. PMID:10304293
Healthy lifestyle beliefs	Personal Beliefs Scale Score range = 10–50 Higher scores indicate higher beliefs	Above 0.80	Melnyk, B. M., Kelly, S., & Tan, A. (2021). Psychometric properties of the Healthy Lifestyle Beliefs Scale for adolescents. *Journal of Pediatric Health Care*, *35*(3), 285–291. https://doi.org/10.1016/j.pedhc.2020.11.002
Healthy lifestyle behaviors	Healthy Lifestyle Behaviors Scale Score range = 16–80 Higher scores indicate higher healthy lifestyle behaviors	0.80 and above	Melnyk, B. M., Jacobson, D., Kelly, S., Belyea, M., Shaibi, G., Small, L., O'Haver, J., & Marsiglia, F. F. (2013). Promoting healthy lifestyles in high school adolescents: A randomized controlled trial. *American Journal of Preventive Medicine*, *45*(4), 407–415. https://doi.org/10.1016/j.amepre.2013.05.013
Mattering	Single‐item Likert‐type question Score range = 0 (not at all) to 4 (very much so)	Not applicable	Melnyk, B. M., Strait, L. A., Beckett, C., Hsieh, A. P., Messinger, J., & Masciola, R. (2023). The state of mental health, burnout, mattering and perceived wellness culture in Doctorally prepared nursing faculty with implications for action. *Worldviews on Evidence‐Based Nursing*, *20*(2), 142–152. https://doi.org/10.1111/wvn.12632
Resilience	Single‐item Likert‐type question Score range = 0 (not at all) to 4 (very much so)	Not applicable	This question was developed for the current study: “How resilient do you believe you are?”

*Note:* AUC = area under the curve, measures overall diagnostic accuracy where 0.8–0.9 is considered excellent accuracy. For statistical analysis, burnout was considered positive for score ≥ 3, suicidal ideation for score ≥ 1, and other measures treated as continuous variables.

Abbreviations: GAD‐7 = Generalized Anxiety Disorder‐7; JSS = Job Satisfaction Scale; PC‐PTSD‐5 = The Primary Care PTSD Screen for DSM‐5; PHQ‐8 = Patient Health Questionnaire‐8; PHQ‐9 = Patient Health Questionnaire‐9.

### 2.4 Data Analysis

Descriptive statistics were used to summarize sample characteristics and prevalence of having suicidal ideation, overall and by sample characteristics. Pearson correlation tests were used to examine pair‐wise correlations among main variables, including suicidal ideation, shift hours, burnout, anxiety, depression, post‐traumatic stress, job satisfaction, mattering, intent to leave, resilience, healthy lifestyle behaviors, and healthy lifestyle beliefs. Unadjusted logistic regression models were used to examine each factor's associated risk of having suicidal ideation. Due to the high correlations between main variables (e.g., *r* = 0.78 for anxiety and depression), we did not proceed to adjusted analysis to avoid biases from mutual adjustment (or Table 2 fallacy) (Green and Popham 2019; Westreich and Greenland 2013). SAS 9.4 was used for all analyses (SAS Institute Inc., Cary, North Carolina).

**TABLE 2:** Descriptive statistics of sample characteristics (*N* = 499).

Characteristics	Mean (SD) or *N* (%)
Age, mean (SD)	42.0 (11.3)
Gender, *N* (%)^a^	
Female	459 (92.0%)
Male	31 (6.2%)
Race/Ethnicity, *N* (%)^a^	
Non‐Hispanic White	416 (83.2%)
Non‐Hispanic Black	23 (4.6%)
Hispanics	29 (5.8%)
Other	29 (5.8%)
Marital status, *N* (%)^a^	
Never married	106 (21.2%)
Currently married	296 (59.3%)
Separated/Divorced/Widowed	83 (16.6%)
Role, *N* (%)^a^	
Staff	292 (58.5)
Manager/Supervisor	91 (18.2)
Advanced practice nurse	10 (2.0)
Licensed nurse	23 (4.6)
Other	70 (14.0)
Shift length, *N* (%)^a^	
4 h	3 (0.6%)
8 h	165 (33.1%)
10 h	91 (18.2%)
12 h	216 (43.2%)
Other	23 (4.6%)
Burnout, *N* (%)^a^	
No	168 (33.6%)
Yes	329 (65.8%)
GAD‐7, mean (SD)	8.6 (5.5)
PHQ‐8, mean (SD)	8.6 (5.7)
PC‐PTSD‐5, mean (SD)	1.4 (1.8)
Job satisfaction, mean (SD)	24.1 (5.7)
Workplace mattering, mean (SD)	2.1 (1.2)
Intent to leave, mean (SD)	1.3 (1.4)
Resilience, mean (SD)	2.8 (0.9)
Healthy lifestyle behaviors, mean (SD)	52.5 (9.8)
Healthy lifestyle beliefs, mean (SD)	35.3 (6.3)

Abbreviations: GAD‐7 = Generalized Anxiety Disorder‐7; PC‐PTSD‐5 = The Primary Care PTSD Screen for DSM‐5; PHQ‐8 = Patient Health Questionnaire‐8.

^a^ The categories may not add up to total due to missing values. The *n* (%) of missing data were 9 (1.8%) for gender, 3 (0.6%) for race/ethnicity, 14 (2.8%) for marital status, 1 (0.2%) for shift hours, and 3 (0.6%) for burnout status.

## 3 Results

### 3.1 Participants

Of the nurses contacted for recruitment, 501 eligible nurses completed baseline data collection. Two were excluded as they did not provide a response to the PHQ‐9, item 9 regarding suicidality, leaving *n* = 499. Most participants were female (92.0%), staff nurses (58.5%), and Non‐Hispanic White (83.2%) with a mean age of 42 years (Table 2). Just under half (43.2%) worked 12‐h shifts.

### 3.2 Descriptive Data

The majority of nurses (65.8%) reported feelings of burnout. Although mean scores on the GAD‐7 (8.6) and PHQ‐8 (8.6) reflected mild anxiety and mild depression, 22.4% (*n* = 112) screened positive for moderate anxiety, 17.2% (*n* = 86) for severe anxiety, 20.6% (*n* = 103) for moderate depression, 12.6% (*n* = 63) for moderately severe depression, and 5.6% (*n* = 28) for severe depression. More than half of the nurses (59.6%, *n* = 298) had low perceptions of mattering to their workplace, 28% (*n* = 140) had low job satisfaction, and 23.6% (*n* = 118) had high intentions of leaving their job within the next year.

### 3.3 Correlations Between the Main Variables and Suicidal Ideation

Table 3 displays the correlations among the main variables with suicidal ideation. Suicidal ideation was significantly and positively correlated with burnout (0.20, *p* < 0.001), anxiety (0.31, *p* < 0.001), depression without suicidality (PHQ‐8) score (0.41, *p* < 0.001), PTSD (0.23, *p* < 0.001), intent to leave job in the next year (0.18, *p* < 0.001), and shift length (0.09, *p* < 0.05). Significant negative correlations were observed among suicidal ideation and job satisfaction (−0.12, *p* < 0.01), perceptions of mattering to the workplace (−0.20, *p* < 0.001), resilience (0.21, *p* < 0.001), healthy lifestyle behaviors (−0.20, *p* < 0.001), and healthy lifestyle beliefs (−0.34, *p* < 0.001).

**TABLE 3:** Correlations among main variables.

	Shift hours	Burnout	GAD‐7	PHQ‐8	PTSD‐5	Job satisfaction	Workplace mattering	Intent to leave	Resilience	HLBV	HPB
Suicidal ideation	0.09*	0.20***	0.32***	0.41***	0.23***	−0.12**	−0.20***	0.18***	−0.21***	−0.20***	−0.34***
Shift hours	—	0.08	0.06	0.03	0.02	−0.04	−0.15**	0.02	−0.01	−0.04	−0.03
Burnout	—	—	0.50***	0.50***	0.27***	−0.57***	−0.32***	0.37***	−0.14**	−0.26***	−0.42***
GAD‐7 score	—	—	—	0.78***	0.42***	−0.30***	−0.33***	0.23***	−0.27***	−0.27***	−0.50***
PHQ‐8 score	—	—	—	—	0.45***	−0.29***	−0.31***	0.15**	−0.27***	−0.46***	−0.59***
PC‐PTSD‐5 score	—	—	—	—	—	−0.13**	−0.18***	0.05	−0.07	−0.13**	−0.23***
Job satisfaction score	—	—	—	—	—	—	0.42***	−0.48***	0.14**	0.20***	0.31***
Workplace mattering	—	—	—	—	—	—	—	−0.32***	0.25***	0.23***	0.36***
Intent to leave job next year	—	—	—	—	—	—	—		−0.05	−0.05	−0.16***
Resilience	—	—	—	—	—	—	—	—	—	0.24***	0.47***
HLBV	—	—	—	—	—	—	—	—	—	—	0.62***

*Note:* Suicidal ideation was measured with item 9 on the Patient Health Questionnaire‐9.

Abbreviations: GAD‐7 = Generalized Anxiety Disorder‐7; HLBV = Healthy Lifestyle Behaviors Scale; HPB = Personal Beliefs Scale; PC‐PTSD‐5 = The Primary Care PTSD Screen for DSM‐5; PHQ‐8 = Patient Health Questionnaire‐8.

* *p* < 0.05.

** *p* < 0.01.

*** *p* < 0.001.

### 3.4 Factors Associated With the Risk of Suicidal Ideation

Unadjusted logistic regressions were performed to examine factors associated with suicidal ideation in nurses (Table 4).

**TABLE 4:** Factors associated with the risk of having suicidal ideation.

	Having suicide ideation, mean (SD) or *N* (row %)		OR (95% CI) of having suicide ideation^a^
	No	Yes	
All	426 (85.2%)	74 (14.8%)	n/a
Age	42.3 (11.4)	40.1 (10.5)	0.98 (0.96, 1.00)
Gender			
Female	395 (85.9%)	65 (14.1%)	Ref
Male	23 (74.2%)	8 (25.8%)	2.11 (0.91, 4.93)
Race/Ethnicity			
Non‐Hispanic White	352 (84.6%)	64 (15.4%)	Ref
Non‐Hispanic Black	20 (87.0%)	3 (13.0%)	0.83 (0.24, 2.86)
Hispanics	25 (86.2%)	4 (13.8%)	0.88 (0.30, 2.61)
Other	26 (89.7%)	3 (10.3%)	0.63 (0.19, 2.16)
Marital status			
Never married	82 (77.4%)	24 (22.6%)	1.94 (1.10, 3.41)*
Currently married	258 (86.9%)	39 (13.1%)	Ref
Separated/Divorced/Widowed	72 (86.7%)	11 (13.3%)	1.01 (0.49, 2.07)
Role			
Staff	238 (81.5%)	54 (18.5%)	3.90 (1.51, 10.08)*
Manager/Supervisor	86 (94.5%)	5 (5.5%)	Ref
Advanced practice nurse	8 (80.0%)	2 (20.0%)	4.30 (0.72, 25.82)
Licensed nurse	18 (78.3%)	5 (21.7%)	4.78 (1.25, 18.24)*
Other	62 (88.6%)	8 (11.4%)	2.22 (0.69, 7.11)
Shift hours			
< 12	248 (88.6%)	32 (11.4%)	Ref
12+	177 (80.8%)	42 (19.2%)	1.84 (1.12, 3.03)*
Burnout			
No	154 (91.7%)	14 (8.3%)	Ref
Yes	269 (81.8%)	60 (18.2%)	2.45 (1.33, 4.54)**
GAD‐7 score	7.9 (5.2)	12.6 (5.2)	1.17 (1.12, 1.23)***
PHQ‐8 score	7.7 (5.3)	14.1 (5.0)	1.22 (1.16, 1.28)***
PC‐PTSD‐5 score	1.2 (1.7)	2.5 (2.0)	1.44 (1.26, 1.65) ***
Job Satisfaction Score	24.3 (5.7)	22.6 (5.8)	0.95 (0.91, 0.99)*
Workplace mattering	2.2 (1.2)	1.5 (1.1)	0.60 (0.48, 0.75)***
Intent to leave job next year	1.3 (1.4)	1.8 (1.5)	1.31 (1.11, 1.55)**
Resilience	2.9 (0.9)	2.3 (1.1)	0.53 (0.41, 0.69)***
Healthy lifestyle behaviors	53.4 (9.6)	47.3 (9.7)	0.94 (0.91, 0.96) ***
Health‐related personal beliefs	36.3 (5.7)	29.7 (6.3)	0.83 (0.80, 0.88) ***

*Note:* Suicidal ideation was measured with item 9 on the Patient Health Questionnaire‐9. Other race/ethnic category created because additional individual categories were too small to analyze separately or to protect identities.

Abbreviations: GAD‐7 = Generalized Anxiety Disorder‐7; PC‐PTSD‐5 = The Primary Care PTSD Screen for DSM‐5; PHQ‐8 = Patient Health Questionnaire‐8.

^a^ Estimated from unadjusted logistic regression model.

* *p* < 0.05.

** *p* < 0.01.

*** *p* < 0.001.

#### 3.4.1 Demographic Factors

Compared to nurses who were currently married, nurses who were never married had significantly higher odds of suicidal ideation (OR = 1.94, 95% CI [1.10, 3.41], *p* < 0.05). Compared to nurses in manager/supervisor roles, other nurses had higher odds of suicidal ideation (ORs = 2.22–4.78). No significant associations were found for age, gender, or race.

#### 3.4.2 Mental Health, Healthy Lifestyle Behaviors, and Health‐Related Personal Beliefs Factors

Compared to nurses without burnout, nurses with burnout had significantly higher odds of suicidal ideation (OR = 2.45, 95% CI [1.33, 4.54], *p* < 0.01). Similar findings for increased odds of suicidal ideation were observed for nurses with anxiety (OR = 1.17, 95% CI [1.12, 1.23], *p* < 0.001), depression (OR = 1.22, 95% CI [1.16, 1.28], *p* < 0.001), and PTSD (OR = 1.44, 95% CI [1.26, 1.65], *p* < 0.001) when compared to nurses without these mental health outcomes. Resilience, healthy lifestyle behaviors, and health‐related personal beliefs were found to be protective factors against suicidal ideation. Specifically, for each one‐point increase in resilience, the odds of having suicidal ideation decreased by 47% (OR = 0.53, 95% CI [0.41, 0.69], *p* < 0.001).

#### 3.4.3 Workplace Factors

Compared to nurses with shift lengths < 12, nurses with shift lengths ≥ 12 h had significantly higher odds of suicidal ideation (OR = 1.84, 95% CI [1.12, 3.03], *p* < 0.05). A similar significant finding was observed for nurses with an intent to leave their job in the next year (OR = 1.31, 95% CI [1.11, 1.55], *p* < 0.01) when compared to nurses with no intention to leave their job. Higher job satisfaction (OR = 0.95, 95% CI [0.91, 0.99], *p* < 0.05) and higher perceptions of mattering to the workplace (OR = 0.60, 95% CI [0.48, 0.75], *p* < 0.001) were protective factors against suicidal ideation.

## 4 Discussion

Among nurses identified with moderate‐to‐high risk for adverse mental health outcomes, this study detected significant associations among suicidal ideation and burnout, anxiety, depression without suicidality, post‐traumatic stress, low job satisfaction, low perceptions of workplace mattering, and intent to leave their current job within the next year. These associations represent opportunities for suicide prevention in terms of mental health screening, treatment, and modifiable workplace factors.

### 4.1 Relationships Among Burnout, Depression, and Suicidal Ideation

It has previously been reported in a national survey of 7378 nurses that approximately 1 in 18 nurses had suicidal thoughts over the past year (Kelsey et al. 2021). Burnout and depression were found to be independent predictors of suicidal thoughts (Kelsey et al. 2021; Zisook et al. 2024). Our study further confirms that burnout as well as depression are independently associated with suicidality among nurses. When nurses are burned out and/or depressed, healthcare quality and safety also are compromised, as findings in prior studies have indicated an association between these variables and preventable medical errors (Li et al. 2024; Melnyk et al. 2018, 2021; Melnyk, Hsieh, et al. 2023).

### 4.2 Shift Length

Nurses express a preference for 12‐h shift lengths as they perceive that it improves work‐life balance and patient care (Haller et al. 2020). However, it is critical that nurses understand the negative impact that extended shift lengths have on their mental and physical health in addition to patient outcomes. Findings in the current study establish a significant correlation, though a weak association, between shift length and suicidal ideation. Specifically, nurses working 12 or more hours per shift had 1.84 higher odds of experiencing suicidal ideation than nurses working < 12‐h shifts. When nurses work shifts that are 12 or more hours in length, they are more likely to have poor mental and physical health as well as experience burnout (Melnyk et al. 2022). Nurses working 12‐h night shifts can also have impaired driving related to fatigue, increasing their risk of collision on their drive home (James and James 2023). In addition, patients can also be negatively impacted by nurse shift length. In a systematic review including 13 studies, more than half of the studies reported an increase in errors when nurses worked more than 12 h in a single shift (Clendon and Gibbons 2015). These errors included increased frequency in adverse events, central‐line associated bloodstream infections, and care left undone.

### 4.3 Mattering

This study established a significant negative association between suicidal ideation and perceptions of workplace mattering. Specifically, nurses who felt like they mattered to their workplace had 40% lower odds of experiencing suicidal ideation compared to nurses with low perceptions of mattering. Mattering, a construct from social psychology, is defined as “an individual's perception that he or she makes a difference in the lives of others and is significant in the world” (Haizlip et al. 2020, 268). In order to feel fulfilled in one's work, it is vital that nurses feel like the work they do matters to those around them. A sense of mattering cannot simply be accomplished by internally knowing they do good work, as the contribution of hearing others' positive feedback to their work plays an equally important role. In a cross‐sectional study with 324 nurses, higher perceptions of mattering at work were significantly correlated with lower rates of burnout (Haizlip et al. 2020). This finding is further corroborated by Melnyk, Chenot, et al. (2024) and Melnyk, Strait, et al. (2023). Mattering at the workplace appears to play an important role in alleviating both burnout and suicidal ideation. The current study suggests that the inverse may also be true, that not mattering is associated with and may contribute to burnout and suicidal ideation.

### 4.4 Implications for Action

Findings from this study have several important implications for healthcare executives and nursing leaders. An investment in routine screening for burnout and mental health issues in the nursing and healthcare workforce is imperative, along with the provision of mental health counselors and evidence‐based interventions, such as mindfulness (Melnyk et al. 2020; Ramachandran et al. 2023) and cognitive‐behavioral therapy‐based programs like MINDBODYSTRONG, which can also be used preventively to build mental health resiliency (Sampson et al. 2019, 2020).

Mental health stigma must be addressed so that nurses feel comfortable seeking help for mental health concerns without fearing loss of licensure. Most states ask invasive mental health questions on their nurse licensure applications, such as “Within the past 5 years, have you been or are you currently being treated, or on medication for, any mental or emotional illness which may impair or interfere with your ability to practice safely and in a competent and professional manner?” (Melnyk, Holod, et al. 2023). State boards of nursing in these states should be contacted and urged to remove these questions. Health systems and hospitals also need to remove these questions from credentialling applications.

System issues known to be root causes of burnout must be fixed, such as short staffing, problems with the electronic health record, and unnecessary bureaucratic tasks. Leaders must show sincere recognition for their employees' efforts, recognize excellent work, communicate regularly that they matter, and eliminate 12‐h shifts. A sustainable culture of well‐being, led by a chief wellness officer, has been shown to improve the mental health and healthy lifestyle behaviors of nurses and the healthcare workforce and may be one replicable strategy to effect positive change (Melnyk 2023; Melnyk et al. 2022; Melnyk, Chenot, et al. 2024; Melnyk, Hsieh, et al. 2023; Melnyk, Strait, et al. 2023).

### 4.5 Implications for Future Research

Future research is needed to study burnout reduction strategies with the intent to reduce depression and suicidality among nurses. Our study was an evaluation of baseline data for an in‐progress RCT that is investigating a digitalized version of a cognitive‐behavioral skills‐building program (i.e., MINDBODYSTRONG) (Melnyk, Davidson, et al. 2024) combined with an interactive screening program for emotional distress and suicidal ideation compared to the screening program alone. The digitalized intervention is designed to improve the ability to cope with stressors and optimize mental health and well‐being, similar to the manualized version of MINDBODYSTRONG, which has been shown to decrease depression and anxiety as well as improve job satisfaction in early career nurses (Sampson et al. 2019, 2020).

Leadership studies are needed to test interventions designed to improve the perception of mattering in the workplace that also include mental health outcomes of the nursing workforce in addition to traditional measures of job satisfaction and intention to stay.

### 4.6 Limitations

The AFSP ISP is used for screening purposes and is an assessment tool designed to detect people at risk of adverse mental health outcomes, including suicide. It is not a validated diagnostic tool (AFSP, n.d.; Zisook et al. 2015, 2024).

In some cases, participants imposed a delay (up to 94 days) between signing the original study consent and completion of the validated tools, during which time the mental health of the participant may have changed. Most participants (92%) completed the baseline assessment survey within 10 days of providing consent to participate in the study.

The sampling process was originally designed to recruit a national random sample through the Healthy Nurse Healthy Nation American Nurses Association membership. Since this sampling strategy did not reach the desired enrollment rates, we completed enrollment through large healthcare organizations located in each region of the United States and an additional professional nursing organization. Lastly, caution should be taken with the interpretation of associations between suicidal ideation, shift length, and job satisfaction as the correlations were < 0.2, indicating weak associations. Statistical significance was obtained because the sample size was large and there was substantial power.

## 5 Linking Evidence to Action

- Addressing burnout as a potential precursor to treatable mental health disorders is critical and not to be viewed as just an operational retention issue.
- Leaders need to invest in mental health screening, provide evidence‐based mental health and resiliency building intervention programs and resources, fix system issues that are at the root cause of burnout, eliminate 12‐h shifts, and implement tactics to ensure that employees feel that they matter.
- Inclusion efforts should focus on hearing the voices of nurses and appreciating them, prioritizing their ideas for change, and recognizing those who contribute to improving culture and caring practices.

## 6 Conclusion

Prior research has established that the nursing workforce is at increased risk for adverse mental health outcomes and suicide. The current study establishes significant associations among suicidal ideation, burnout, anxiety, depression, post‐traumatic stress, and workplace factors, specifically low job satisfaction, low perceptions of mattering, and intent to leave their current job within the next year in high‐risk nurses. There is an urgent need for policies and evidence‐based interventions to address these issues.

## Conflicts of Interest

Dr. Bernadette Mazurek Melnyk is the creator of the MINDBODYSTRONG program and has a company entitled COPE2Thrive LLC, which disseminates it along with the original and other adapted versions of this program entitled Creating Opportunities for Personal Empowerment (COPE). The remaining authors declare no competing interests.
